# Bone-Modifying Agents in Patients With High-Risk Metastatic Castration-Sensitive Prostate Cancer Treated With Abiraterone Acetate

**DOI:** 10.1001/jamanetworkopen.2024.2467

**Published:** 2024-03-15

**Authors:** Wataru Fukuokaya, Keiichiro Mori, Fumihiko Urabe, Taro Igarashi, Takafumi Yanagisawa, Shunsuke Tsuzuki, Mariko Honda, Kenta Miki, Takahiro Kimura

**Affiliations:** 1 Department of Urology, The Jikei University School of Medicine, Tokyo, Japan

## Abstract

**Importance:**

The association between the use of bone-modifying agents (BMAs) and the outcomes among patients with metastatic castration-sensitive prostate cancer (mCSPC) treated with abiraterone acetate plus prednisone (AAP) remains unclear.

**Objective:**

To investigate the association between BMA use and the outcomes of patients with mCSPC receiving AAP.

**Design, Setting, and Participants:**

In this cohort study, a post hoc analysis of individual participant data from the LATITUDE trial was performed. The LATITUDE trial, a phase 3 randomized clinical trial, aimed to assess the efficacy of AAP and androgen deprivation therapy (ADT) vs dual-placebo and ADT in patients with high-risk mCSPC (data cutoff, August 15, 2018). Eligible patients had newly diagnosed prostate cancer with metastases and at least 2 of 3 high-risk factors (Gleason score ≥8, presence of ≥3 lesions on bone scan, or presence of measurable visceral metastasis). The trial was conducted at 235 sites in 34 countries. Data for the present study were evaluated from July 18 to September 23, 2023.

**Exposures:**

Use of BMAs was defined as the administration of bisphosphonates and denosumab within 90 days before and after randomization.

**Main Outcomes and Measures:**

The primary outcomes were time to skeletal-related events (SREs) and overall survival (OS). An SRE was defined as a clinical or pathological fracture, spinal cord compression, palliative radiation to bone, or surgery involving bone. Differences in these outcomes were examined using the restricted mean survival time from inverse probability of treatment weighting–adjusted Kaplan-Meier curves, estimated until the last event was observed (longest time observed, 63.9 months). Treatment × covariate interactions were analyzed using weighted Cox proportional hazards regression models for the total cohort.

**Results:**

In the total cohort of 1199 patients (956 [79.7%] younger than 75 years), 597 (49.8%) received AAP and ADT, including 474 (79.4%) younger than 75 years and 384 (64.3%) with more than 10 bone metastases (AAP cohort); 602 (50.2%) were treated with dual placebo and ADT, including 482 (80.1%) younger than 75 years and 377 (62.6%) with more than 10 bone metastases (ADT cohort). In the AAP cohort, 132 patients (22.1%) received BMAs, while in the ADT cohort, 131 (21.8%) did. Zoledronic acid was the most frequently administered BMA in both the AAP (93 [70.5%]) and the ADT (88 [67.2%]) cohorts. During the median follow-up of 51.8 (IQR, 47.2-57.0) months in the AAP cohort, BMA use was associated with a longer time to SRE (difference, 7.8 [95% CI, 4.2-11.3] months) but not with OS (difference, 1.6 [95% CI, −2.5 to 5.8] months). In the ADT cohort, BMA use was associated with both time to SRE (difference, 9.3 [95% CI, 5.2-13.3] months) and OS (difference, 5.5 [95% CI, 3.2-9.8] months). No evidence was found that the outcomes of BMA varied by AAP or ADT (hazard ratio for time to SRE, 0.99 [95% CI, 0.48-2.08]; *P* = .99 for interaction; hazard ratio for OS, 1.31 [95% CI, 0.88-1.96]; *P* = .18 for interaction).

**Conclusions and Relevance:**

The findings of this cohort study suggest that use of BMAs was associated with a longer time to SRE in patients with high-risk mCSPC treated with ADT, with or without AAP, suggesting that BMA use might provide benefits to this population.

## Key Points

**Question:**

Is the use of bone-modifying agents (BMAs) such as bisphosphonates and denosumab associated with the outcomes among patients with metastatic castration-sensitive prostate cancer (mCSPC) treated with abiraterone acetate and prednisone?

**Findings:**

In this cohort study using a post hoc analysis of data from the LATITUDE trial, BMA use was associated with a longer time to skeletal-related events but not with overall survival in patients with high-risk mCSPC receiving androgen deprivation therapy with abiraterone acetate and prednisone.

**Meaning:**

These findings suggest that BMA use is associated with a longer time to skeletal-related events in patients with high-risk mCSPC receiving abiraterone acetate and prednisone.

## Teaser

This cohort study uses data from the LATITUDE trial to investigate the association of bone-modifying agent use with time to skeletal-related events and overall survival in patients with high-risk metastatic castration-sensitive prostate cancer treated with abiraterone acetate and prednisone.

## Introduction

Metastatic prostate cancer is a lethal disease, with a 5-year overall survival (OS) rate of 29.8% in patients with metastatic, castration-sensitive prostate cancer (mCSPC).^1^ One of the major complications affecting the quality of life of these patients is skeletal-related events (SREs). Such events may include pathologic fractures, spinal cord compression, palliative radiation, or surgery to the bone. Between 30% and 50% of patients with metastatic prostate cancer encounter at least 1 SRE during their disease progression.^2^

Bone-modifying agents (BMAs) such as bisphosphonates and denosumab are effective in improving time to SRE in patients with metastatic castration-resistant prostate cancer. Consequently, current guidelines recommend their use for this population.^3,4,5,6^ By contrast, a meta-analysis of data from 3 randomized clinical trials (RCTs)^7^ did not find evidence that zoledronic acid, a third-generation bisphosphonate, improved OS in patients with mCSPC. Additionally, 2 RCTs^8,9^ provided conflicting results regarding time to SRE. Consequently, current guidelines do not suggest the use of bisphosphonates for mCSPC.^5,6^ In a recent advanced prostate cancer consensus conference,^10^ only 10% to 20% of the panelists indicated that they routinely recommend initiating BMA use for patients with mCSPC treated with androgen deprivation therapy (ADT), with or without androgen receptor–signaling inhibitors, to prevent treatment-induced bone loss.

Abiraterone acetate was the first androgen receptor–signaling inhibitor to receive regulatory approval for high-risk mCSPC, and a recent study^11^ showed that a regimen combining abiraterone acetate and prednisone (AAP) was the most commonly used androgen receptor–signaling inhibitor among patients with mCSPC. In managing mCSPC, abiraterone acetate is coadministered with prednisone at a dosage of 5 mg, potentially resulting in increased bone fragility.^12^ While 2 RCTs have shown that AAP improved time to SRE in patients with mCSPC,^13,14^ the association between BMA use and time to SRE for these patients remains unclear. Thus, using data from the LATITUDE trial, we investigated the association of BMA use with time to SRE and survival in patients with high-risk mCSPC treated with AAP. Additionally, this association was evaluated in patients treated with ADT using the same data.

## Methods

The Certified Review Board of The Jikei University exempted this cohort study from review and consent, as it used anonymized, publicly available clinical trial data. We followed the Strengthening the Reporting of Observational Studies in Epidemiology (STROBE) reporting guideline.

### Data Source

We conducted a post hoc analysis using individual participant data from the LATITUDE trial (data cutoff, August 15, 2018).^15^ The LATITUDE trial was an international, double-blind, placebo-controlled, phase 3 RCT designed to assess the efficacy and safety of AAP combined with ADT in patients with newly diagnosed (<3 months prior to randomization) mCSPC.^14^ The trial was conducted at 235 sites in 34 countries. This study included patients with high-risk mCSPC, and participants needed to have at least 2 of the 3 subsequent high-risk prognostic factors: a Gleason score of 8 or more, a minimum of 3 bone lesions, or the presence of measurable visceral metastasis.^16^ Between February 12, 2013, and December 11, 2014, 1209 patients underwent eligibility assessment, 10 of whom were found ineligible due to violations at the study sites. As a result, 1199 patients were randomized at a 1:1 ratio to receive either abiraterone acetate (1000 mg/d, administered once orally) plus prednisolone (5 mg/d) with ADT (the AAP cohort [n = 597]) or a combination of dual placebo with ADT (the ADT cohort [n = 602]). This study used the final dataset, with evaluations conducted from July 18 to September 23, 2023.^16^

### Exposures and Variable Definitions

Use of BMAs was defined as bisphosphonate or denosumab administration within a 90-day window, both before and after randomization. We selected this time frame because the study included patients diagnosed within 3 months prior to randomization, and within the 90 days following randomization, most patients showed no radiographic progression, as depicted by the Kaplan-Meier curves in the prior report.^14^ Other covariates included age at the time of randomization, Eastern Cooperative Oncology Group performance status, baseline prostate-specific antigen (PSA) levels, pain as measured by the Brief Pain Inventory–Short Form 3, Gleason grade group, number of bone metastases, evidence of visceral metastasis, evidence of liver metastasis, baseline hemoglobin concentration, baseline serum creatinine level, and baseline lactate dehydrogenase (LDH) level. Considering that BMAs are frequently prescribed to prevent osteoporosis, we also examined the risk factors for osteoporosis, such as concomitant proton pump inhibitor (PPI) use and a history of diabetes.^17,18^ Concomitant PPI use was defined as any PPI use within a window of 30 days before and after randomization based on the prior analyses.^19,20^

### Outcomes

The primary outcomes evaluated were time to the first symptomatic SRE and OS. Both outcomes were defined according to the trial protocol.^14^ Time to SRE was calculated from the date of randomization to the earliest occurrence of a clinical or pathological fracture, spinal cord compression, palliative radiation to the bone, or surgery to the bone. In this study, these outcomes were assessed between randomization and the last follow-up time (either event or censored) (longest observation, 63.9 months).

### Statistical Analysis

Descriptive statistics were used to compare the baseline characteristics of BMA users and nonusers. Because of the skewed distribution of raw laboratory data, a natural logarithmic transformation was applied to the baseline levels of PSA and LDH to achieve a more normal distribution. The missing baseline data were handled based on a random forest imputation algorithm.^21^ Propensity scores, which represent the probability of being a BMA user, were calculated based on a multivariable logistic regression model. The covariates included age at randomization (≥75 or <75 years), Eastern Cooperative Oncology Group performance status (2 or 0-1), baseline PSA (log-transformed), Brief Pain Inventory–Short Form 3 score (≥4 or 1-3), Gleason grade group (5 or 1-4), number of bone metastases (≥10 or <10), evidence of visceral metastasis (presence or absence), evidence of liver metastasis (presence or absence), baseline hemoglobin concentration (continuous), baseline serum creatinine concentration (continuous), and baseline LDH level (log-transformed). The distributions of propensity scores were visualized via Kernel density estimation. Stabilized inverse probability of treatment weighting (IPTW) based on propensity scores was applied to balance the baseline characteristics between BMA users and nonusers. After weighting, standardized mean differences (SMDs) were calculated to quantify balance between the groups, with an SMD less than 0.1 for each covariate indicating adequate balance.^22^ The time-to-event distributions of the crude and weighted populations were estimated based on the Kaplan-Meier plot. The between-group differences in time-to-event outcomes were analyzed using restricted mean survival times (RMSTs) based on the IPTW-adjusted Kaplan-Meier plots. The RMST is the mean event-free time until a milestone point, a numerical expression of the area under the Kaplan-Meier plot.^23^ In this study, RMSTs were estimated until the last event was observed. Additionally, the changes in the difference in RMSTs throughout the follow-up period were visualized in a sensitivity analysis.^24^ To further assess the association between BMA use and time to SRE, we evaluated the differences in RMSTs between BMA users and nonusers for each element that defines an SRE, including time to palliative radiation to the bone, time to clinical fracture, time to spinal cord compression, and time to surgery to the bone. Hazard ratios (HRs) and the corresponding 95% CIs were estimated using IPTW-adjusted Cox proportional hazards regression models.

Furthermore, we expanded this analysis to the data on all participants in the LATITUDE trial (N = 1199) to test whether the effect of BMAs varied by treatment arm (AAP or ADT). In this analysis, covariate balances were adjusted using IPTWs calculated based on propensity scores estimated by a multivariable logistic regression model with the covariate used in the AAP cohort plus the treatment arm (AAP or ADT). The IPTW-adjusted Cox proportional hazards regression models were applied to assess BMA use × treatment arm interaction on time to SRE and OS. Additionally, we investigated how the effects of BMA use on time to SRE varied by baseline characteristics based on interaction terms within IPTW-adjusted Cox proportional hazards regression models. Given the exploratory nature of this post hoc analysis, no adjustment for multiple testing was conducted.

All statistical tests were 2 sided, and *P* < .05 indicated statistical significance. All statistical analyses were performed using R, version 4.3.0 (R Project for Statistical Computing).

## Results

### Baseline Characteristics

The total cohort of 1199 patients consisted of 956 (79.7%) who were younger than 75 years, 1159 (96.7%) with an Eastern Cooperative Oncology Group performance status of 0 to 1, 228 (19.0%) presenting evidence of visceral metastasis, and 761 (63.5%) with more than 10 bone metastases. The most frequently missing data pertained to the Brief Pain Inventory–Short Form 3, absent for 50 of 1199 patients (4.2%). Among the 597 patients in the AAP cohort, 474 (79.4%) were younger than 75 years and 384 (64.3%) had more than 10 bone metastases. Among the 602 patients in the ADT cohort, 482 (80.1%) were younger than 75 years and 377 (62.6%) had more than 10 bone metastases. During a median follow-up of 51.8 (IQR, 47.2-57.0) months, 3 patients (0.5%) from the AAP cohort and 2 (0.3%) from the ADT cohort were lost to follow-up. The study flow is depicted in eFigure 1 in Supplement 1. Of the total cohort, 132 patients (22.1%) in the AAP cohort and 131 (21.8%) in the ADT cohort received BMAs within 90 days, both before and after randomization (eFigure 2 in Supplement 1). The details of the BMAs are detailed in eTable 1 in Supplement 2. Zoledronic acid was the most frequently administered BMA, with 93 of 132 patients (70.5%) patients in the AAP cohort and 88 of 131 (67.2%) in the ADT cohort.

There was no appreciable difference in baseline characteristics before and after imputation (eTable 2 in Supplement 2). In the total cohort, 110 patients (9.2%) were using concomitant PPI, 180 (15.0%) had a history of diabetes, 761 (63.5%) presented with 10 or more bone metastases, and 228 (19.0%) had visceral metastases. The Table provides a summary of the baseline characteristics of the AAP and ADT cohorts. After the application of IPTWs, there was a balance in the baseline characteristics between BMA users and nonusers across all cohorts (SMD <0.1 for all) (eTables 3-5 in Supplement 2), and the distributions of the estimated propensity scores were similar between BMA users and nonusers across the 3 cohorts (eFigures 3-5 in Supplement 1). Among SREs that occurred first during the follow-up, the most frequent event was palliative radiation to the bone, occurring in 74 of 132 patients (56.1%) from the AAP cohort and 102 of 150 (68.0%) from the ADT cohort. Further SRE details can be found in eTable 6 in Supplement 2.

**Table.** Baseline Characteristics of the AAP and ADT Cohorts^a^

Characteristic	AAP cohort			ADT cohort		
	All (n = 597)	Nonusers (n = 465)	BMA users (n = 132)	All (n = 602)	Nonusers (n = 471)	BMA users (n = 131)
Age at randomization, y						
≥75	123 (20.6)	99 (21.3)	24 (18.2)	120 (19.9)	96 (20.4)	24 (18.3)
<75	474 (79.4)	366 (78.7)	108 (81.8)	482 (80.1)	375 (79.6)	107 (81.7)
ECOG performance status^b^						
0-1	573 (96.0)	446 (95.9)	127 (96.2)	586 (97.3)	458 (97.2)	128 (97.7)
2	24 (4.0)	19 (4.1)	5 (3.8)	16 (2.7)	13 (2.8)	3 (2.3)
Concomitant PPI use	60 (10.1)	43 (9.2)	17 (12.9)	50 (8.3)	39 (8.3)	11 (8.4)
Diabetes	87 (14.6)	69 (14.8)	18 (13.6)	93 (15.4)	73 (15.5)	20 (15.3)
Baseline PSA level (log-transformed), median (IQR)	3.9 (2.2-5.3)	3.8 (2.0-5.3)	4.0 (3.1-5.6)	3.8 (2.2-5.3)	3.7 (2.2-5.3)	4.0 (2.1-5.7)
Brief Pain Inventory–Short Form 3^c^						
0-3	436 (73.0)	338 (72.7)	98 (74.2)	449 (74.6)	355 (75.4)	94 (71.8)
≥4	161 (27.0)	127 (27.3)	34 (25.8)	153 (25.4)	116 (24.6)	37 (28.2)
Gleason grade group^d^						
1-4	280 (46.9)	213 (45.8)	67 (50.8)	297 (49.3)	232 (49.3)	65 (49.6)
5	317 (53.1)	252 (54.2)	65 (49.2)	305 (50.7)	239 (50.7)	66 (50.4)
No. of bone metastases						
0-10	213 (35.7)	171 (36.8)	42 (31.8)	225 (37.4)	180 (38.2)	45 (34.4)
>10	384 (64.3)	294 (63.2)	90 (68.2)	377 (62.6)	291 (61.8)	86 (65.6)
Evidence of visceral metastasis	114 (19.1)	87 (18.7)	27 (20.5)	114 (18.9)	94 (20.0)	20 (15.3)
Evidence of liver metastasis	32 (5.4)	22 (4.7)	10 (7.6)	30 (5.0)	25 (5.3)	5 (3.8)
Baseline hemoglobin concentration, median (IQR), g/dL	13.2 (12.0-14.3)	13.2 (12.1-14.4)	13.2 (11.9-14.1)	13.3 (12.1-14.4)	13.3 (12.1-14.5)	13.2 (11.8-14.2)
Baseline serum creatinine concentration, median (IQR), mg/dL	0.90 (0.80-1.06)	0.93 (0.80-1.08)	0.89 (0.79-1.03)	0.90 (0.80-1.04)	0.90 (0.80-1.06)	0.88 (0.76-1.01)
Baseline LDH concentration (log-transformed), median (IQR), U/L	5.2 (5.1-5.3)	5.2 (5.1-5.3)	5.2 (5.0-5.3)	5.2 (5.0-5.3)	5.2 (5.0-5.3)	5.2 (5.1-5.3)

Abbreviations: AAP, abiraterone acetate with prednisone; ADT, androgen deprivation therapy; BMA, bone-modifying agent; ECOG, Eastern Cooperative Oncology Group; LDH, lactate dehydrogenase; PPI, proton-pump inhibitor; PSA, prostate-specific antigen.

SI conversion factors: To convert creatinine to μmol/L, multiply by 88.4; hemoglobin to g/L, multiply by 10.0; and LDH to μkat/L, multiply by 0.0167.

^a^ Unless otherwise indicated, data are expressed as No. (%) of patients. Percentages have been rounded and may not total 100.

^b^ Higher scores indicate worse functioning.

^c^ Higher scores indicate greater severity of pain.

^d^ Higher grades indicate more aggressive tumor with a worse prognosis.

### Outcomes Between BMA Users and Nonusers in the AAP Cohort

**Figure 1.**
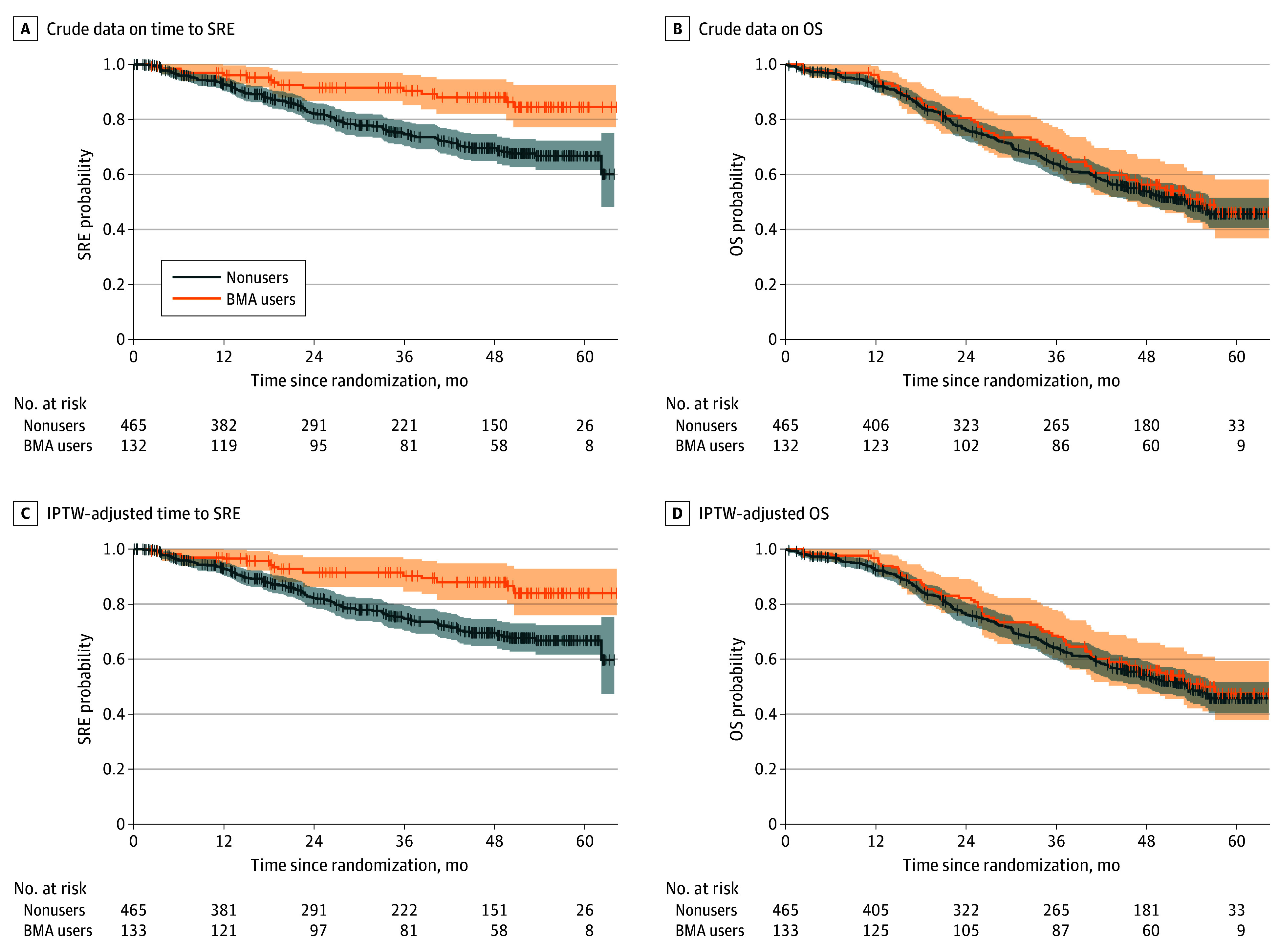
Crude and Inverse Probability of Treatment Weighting (IPTW)–Adjusted Kaplan-Meier Curves Based on Bone-Modifying Agent (BMA) Use in the Abiraterone Acetate Plus Prednisone Cohort Shaded areas indicate 95% CIs. OS indicates overall survival; SRE, skeletal-related event.

**Figure 2.**
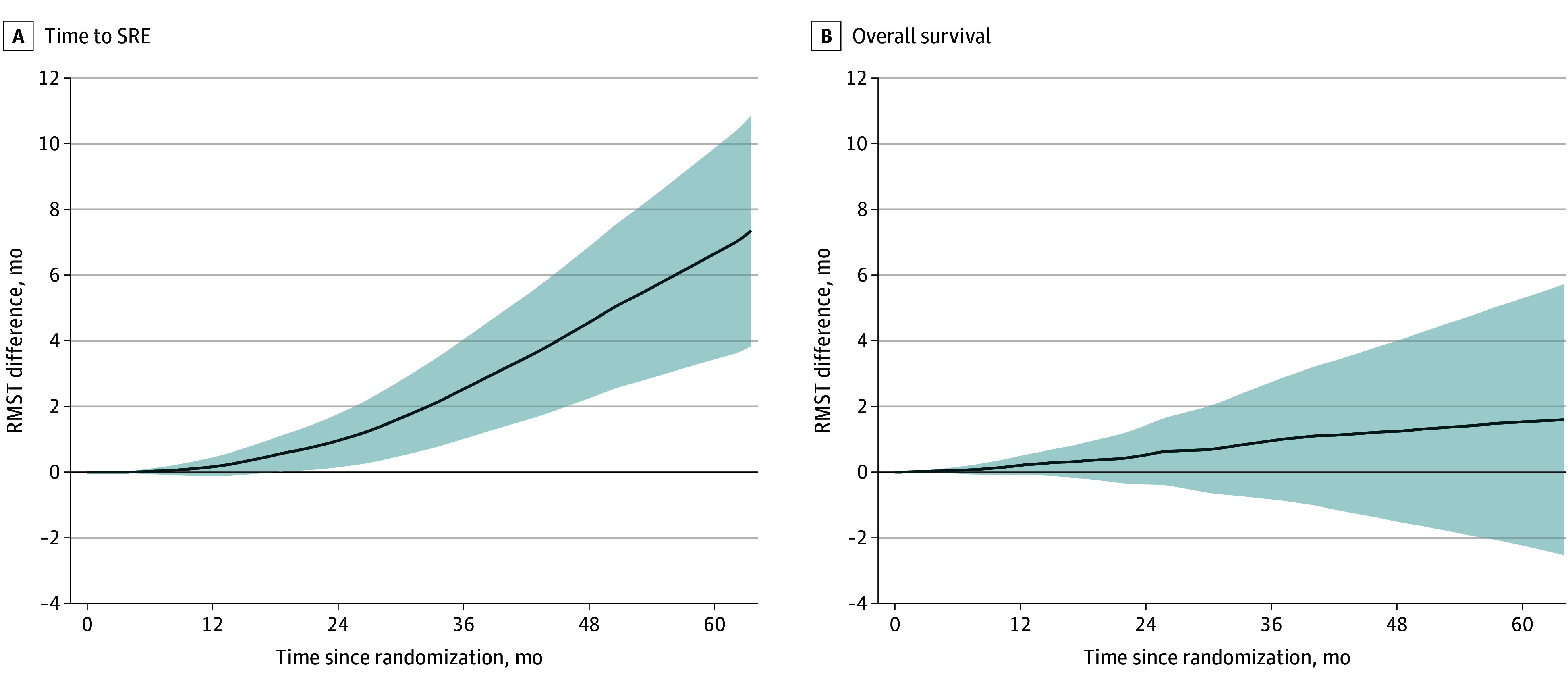
Differences in Restricted Mean Survival Times Between Bone-Modifying Agent Users and Nonusers in the Abiraterone Acetate Plus Prednisone Cohort Differences were calculated as restricted mean survival time for body-modifying agent users – restricted mean survival time for nonusers. A difference greater than 0 indicates longer survival in users, whereas a difference less than 0 indicates longer survival in nonusers. Shaded areas indicate 95% CIs.

Overall, 132 patients (22.1%) in the AAP cohort experienced SREs, and 275 (46.1%) died of any cause. The median time to SRE was unavailable, and the median OS was 53.3 (95% CI, 48.2 to not reached) months. The crude and IPTW-adjusted Kaplan-Meier plots are depicted in Figure 1. The last observed SRE and death due to any cause were observed as long as 63.9 months after randomization. After the application of IPTW, the mean time to SRE was longer in BMA users compared with nonusers (difference, 7.8 [95% CI, 4.2-11.3] months). By contrast, we found no evidence that the difference in RMST for OS between BMA users and nonusers was significant (difference, 1.6 [95% CI, −2.5 to 5.8] months). Sensitivity analyses showed that such associations were consistent throughout the follow-up period (Figure 2). Use of BMA was associated with lower hazard ratios (HRs) for time to SRE (0.40 [95% CI, 0.23-0.70]) but not with OS (0.92 [95% CI, 0.68-1.23]). Moreover, upon examining the association between BMA use and SRE components, we observed longer times to palliative radiation to the bone (difference, 5.1 [95% CI, 2.0-8.3] months) and to clinical fracture (difference, 2.7 [95% CI, 0.7-4.7] months), but there was no evidence of differences in time to spinal cord compression (difference, 0.7 [95% CI, –1.5 to 2.8] months) or surgery to the bone (difference, 0.1 [95% CI, –0.9 to 1.2] months) (eFigures 6 and 7 in Supplement 1). eTable 7 in Supplement 2 lists the results from IPTW-adjusted Cox proportional hazards regression models for SRE components, showing outcomes comparable with the RMST analysis.

### Outcomes Between BMA Users and Nonusers in the ADT Cohort

**Figure 3.**
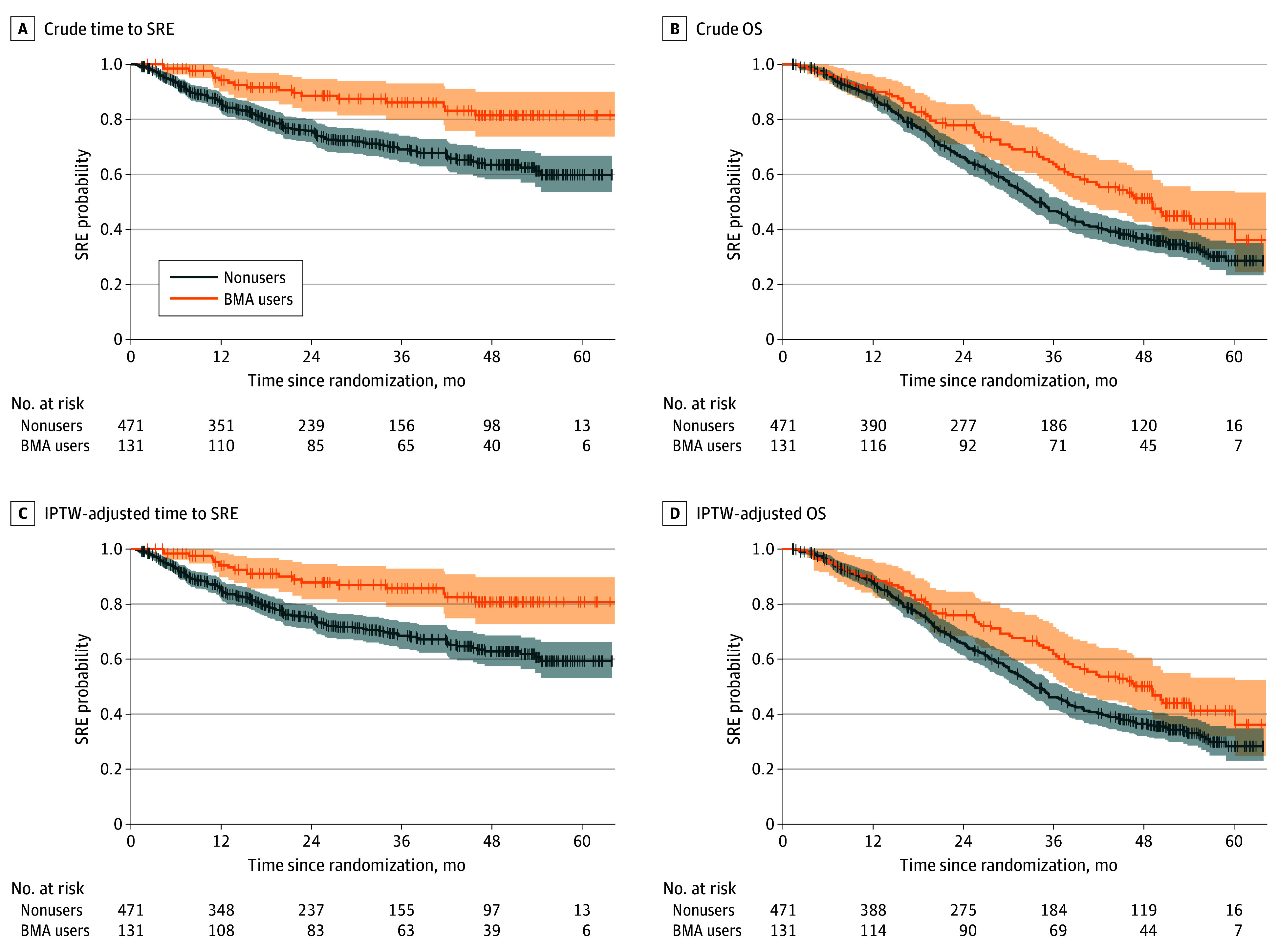
Crude and Inverse Probability of Treatment Weighting (IPTW)–Adjusted Kaplan-Meier Curves Based on Bone-Modifying Agent (BMA) Use in the Androgen Deprivation Therapy Cohort Shaded areas indicate 95% CIs. OS indicates overall survival; SRE, skeletal-related event.

**Figure 4.**
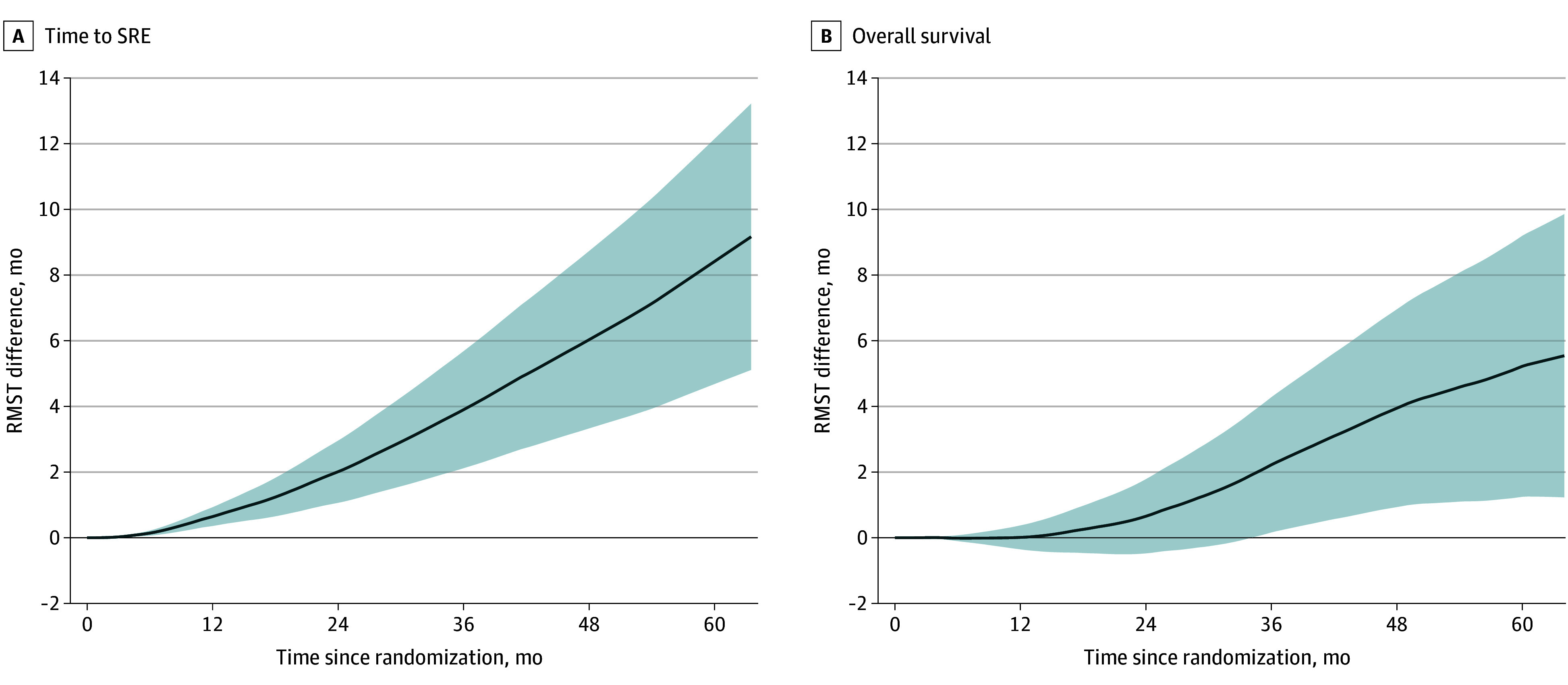
Differences in Restricted Mean Survival Times Between Bone-Modifying Agent Users and Nonusers in the Androgen Deprivation Therapy Cohort Differences were calculated as restricted mean survival time for body-modifying agent users – restricted mean survival time for nonusers. A difference greater than 0 indicates longer survival in users, whereas a difference less than 0 indicates longer survival in nonusers. Shaded areas indicate 95% CIs.

During the follow-up, 150 patients (24.9%) experienced SREs, and 343 (57.0%) died of any causes. Figure 3 illustrates the crude and IPTW-adjusted Kaplan-Meier plots. After the application of IPTW, BMA users had a longer mean time to SRE than nonusers (difference, 9.3 [95% CI, 5.2-13.3] months). Furthermore, the difference in RMST for OS was longer in BMA users than in nonusers (difference, 5.5 [95% CI, 3.2-9.8] months). Sensitivity analysis showed that RMST differences for time to SRE and OS were consistently increased over the follow-up period (Figure 4). Use of BMAs was associated with a higher HR for time to SRE (0.42 [95% CI, 0.26-0.69) and OS (0.72 [95% CI, 0.55-0.95]). Further examination of the association between BMA use and SRE components found longer times to palliative radiation to the bone (difference, 6.4 [95% CI, 2.5-10.4] months), clinical fracture (difference, 2.9 [95% CI, 1.1-4.7] months), and spinal cord compression (difference, 3.7 [95% CI, 2.0-5.3] months). We found no evidence of an association between BMA use and time to surgery to the bone (difference, 0.2 [95% CI, −0.9 to 1.3] months) (eFigures 8 and 9 in Supplement 1). The IPTW-adjusted Cox proportional hazards regression models for SRE components showed similar outcomes to the RMST analysis (eTable 8 in Supplement 2).

### Treatment × Covariate Interaction on Outcomes

We further investigated whether the effect of concomitant use of BMAs varied based on the treatment received (AAP or ADT). The IPTW-adjusted Cox proportional hazards regression models did not show evidence that the effect of BMA on outcomes varied by treatment (HR for time to SRE, 0.99 [95% CI, 0.48-2.08]; *P* = .99 for interaction; HR for OS, 1.31 [95% CI, 0.88-1.96]; *P* = .18 for interaction). Further analyses showed that the effect of BMA on each component of an SRE did not vary by treatment (eTable 8 in Supplement 2). We also analyzed treatment × covariate interactions using IPTW-adjusted Cox proportional hazards regression models. We found no evidence that the effect of BMA use was modified by age, the presence of visceral metastasis, the number of bone metastases, baseline pain scales, and serum creatinine level (eTable 9 in Supplement 2).

## Discussion

This study found that BMA use was associated with a longer time to SRE in patients with high-risk mCSPC treated with ADT, both with and without AAP. Furthermore, the difference in RMST for OS between BMA users and nonusers was similar in the AAP cohort, with a mean difference of 1.6 months over a 63.9-month follow-up period. Similar to the survival curves from 2 RCTs showing the efficacy of zoledronic acid in patients with metastatic prostate cancer,^3,8^ the Kaplan-Meier curves for time to SRE between BMA users and nonusers diverged early in both the AAP and ADT cohorts. On examination of individual SRE components, longer times to palliative radiation to the bone and clinical fracture were observed in both cohorts among those using BMAs. While a longer time to spinal cord compression was noted only in the ADT cohort among BMA users, we did not find evidence showing that the effect of BMA use on time to spinal cord compression varied by treatment (AAP or ADT). Interaction analyses did not show evidence that the effect of BMA use on time to SRE varied by covariates, including age, visceral metastasis, number of bone metastases, baseline pain scales, and baseline serum creatinine level. While the Kaplan-Meier curves for OS based on BMA use appeared to differ between the AAP and ADT cohorts, no evidence was found to indicate a significant difference in the association of BMAs with time to SRE or OS, depending on the treatment (either AAP or ADT). This study showed that 21.9% (263 of 1199) of the patients used BMAs within 90 days before and after randomization. These findings align with data from Surveillance, Epidemiology, and End Results Medicare data in which 18.9% of patients with mCSPC initiated BMA use within 90 days of diagnosis.^25^

While BMAs are recommended for preventing SREs in patients with castration-resistant prostate cancer with bone metastasis,^5,6^ BMA use is not currently recommended for patients with mCSPC based on the results of several RCTs. The CALGB 90202 trial indicated that the addition of zoledronic acid did not increase time to first SRE or OS.^9^ However, that trial has limitations. First, the trial planned to administer zoledronic acid to all participants on developing castration resistance before developing SREs, resulting in masking the potential effect of zoledronic acid on time to SRE. Second, the early termination of the trial limited the study’s power. Additionally, the STAMPEDE (Systemic Therapy in Advancing or Metastatic Prostate Cancer: Evaluation of Drug Efficacy) trial^26^ also reported that the use of zoledronic acid did not improve failure-free survival and OS. However, updated available data from this trial suggested that adding zoledronic acid to ADT was associated with a reduced risk of clinical fracture in mCSPC (HR, 0.36 [95% CI, 0.22-0.57]; *P* < .001) but not in nonmetastatic CSPC (HR, 0.67 [95% CI, 0.32-1.39]; *P* = .28).^26^ Although clinical fracture is part of the composite SREs, these data might support our findings.

### Limitations

This study has some limitations. The results must be viewed in the context of its post hoc analysis design. While IPTW adjustment balanced the baseline covariates between BMA users and nonusers, potential biases could arise from unmeasured confounders and indication biases among physicians. Because of the limited number of patients in BMA users, investigating the differences in the effects of BMAs based on the specific drugs used was not possible. Furthermore, whether the results of this study are applicable to other populations, such as those outside clinical trials, is debatable. These populations may include less-selected, heterogeneous patients and those with greater fragility, as well as those who do not meet the high-risk criteria of the LATITUDE trial.

## Conclusions

The use of BMAs was associated with a longer time to SRE but not with OS in patients with high-risk mCSPC treated with ADT and AAP. Furthermore, the outcomes of BMAs did not vary between patients treated with ADT plus AAP and those receiving ADT alone. Given the exploratory design of this study, these findings must be validated through RCTs.

## Notes

Supplement 1. **eFigure 1.** Study Flow **eFigure 2.** Distributions of the Days Initiating BMA Based on Treatment Received **eFigure 3.** Propensity Score Distributions Between BMA Users and Nonusers Before and After IPTW Adjustment in the Total Cohort **eFigure 4.** Propensity Score Distributions Between BMA Users and Nonusers Before and After IPTW Adjustment in the AAP Cohort **eFigure 5.** Propensity Score Distributions Between BMA Users and Nonusers Before and After IPTW Adjustment in the ADT Cohort **eFigure 6.** IPTW-Adjusted Kaplan-Meier Curves Based on BMA Use in the AAP Cohort **eFigure 7.** Differences in RMSTs Between BMA Users and Nonusers in the AAP Cohort **eFigure 8.** IPTW-Adjusted Kaplan-Meier Curves Based on BMA Use in the ADT Cohort **eFigure 9.** Differences in RMSTs Between BMA Users and Nonusers in the ADT Cohort

Supplement 2. **eTable 1.** Bone-Modifying Agent According to Treatment Arm **eTable 2.** Baseline Characteristics of the Total Cohort **eTable 3.** Covariate Balance Between BMA Users and Nonusers in the Total Cohort **eTable 4.** Covariate Balance Between BMA Users and Nonusers in the AAP Cohort **eTable 5.** Covariate Balance Between BMA Users and Nonusers in the ADT Cohort **eTable 6.** Details of First Skeletal-Related Event According to BMA Use and Treatment Arm **eTable 7.** IPTW-Adjusted Cox Proportional Hazards Regression Analysis for SRE Components (AAP Cohort) **eTable 8.** Treatment-BMA Use Interactions Based on IPTW-Adjusted Cox Proportional Hazards Regression Models **eTable 9.** Covariate-Treatment Interactions Based on IPTW-Adjusted Cox Proportional Hazards Regression Models for Time to SRE (Total Cohort)

Supplement 3.
Data Sharing Statement
