# Supplementary material for: Bone-Modifying Agents in Patients With High-Risk Metastatic Castration-Sensitive Prostate Cancer Treated With Abiraterone Acetate
Source: JAMA Netw Open. 2024 Mar 15;7(3):e242467. doi: 10.1001/jamanetworkopen.2024.2467 (PMC10943414; doi:10.1001/jamanetworkopen.2024.2467)
Supplement: Supplement 1. — eFigure 1. Study Flow eFigure 2. Distributions of the Days Initiating BMA Based on Treatment Received eFigure 3. Propensity Score Distributions Between BMA Users and Nonusers Before and After IPTW Adjustment in the Total Cohort eFigure 4. Propensity Score Distributions Between BMA Users and Nonusers Before and After IPTW Adjustment in the AAP Cohort eFigure 5. Propensity Score Distributions Between BMA Users and Nonusers Before and After IPTW Adjustment in the ADT Cohort eFigure 6. IPTW-Adjusted Kaplan-Meier Curves Based on BMA Use in the AAP Cohort eFigure 7. Differences in RMSTs Between BMA Users and Nonusers in the AAP Cohort eFigure 8. IPTW-Adjusted Kaplan-Meier Curves Based on BMA Use in the ADT Cohort eFigure 9. Differences in RMSTs Between BMA Users and Nonusers in the ADT Cohort [file jamanetwopen-e242467-s001.pdf]

Supplementary Online Content

Fukuokaya W, Mori K, Urabe F, et al. Use of bone-modifying agents in patients with high-risk metastatic castration-sensitive prostate cancer. *JAMA Netw Open*. 2024;7(3):e242467. doi:10.1001/jamanetworkopen.2024.2467

eFigure 1. Study Flow
eFigure 2. Distributions of the Days Initiating BMA Based on Treatment Received
eFigure 3. Propensity Score Distributions Between BMA Users and Nonusers Before and After IPTW Adjustment in the Total Cohort
eFigure 4. Propensity Score Distributions Between BMA Users and Nonusers Before and After IPTW Adjustment in the AAP Cohort
eFigure 5. Propensity Score Distributions Between BMA Users and Nonusers Before and After IPTW Adjustment in the ADT Cohort
eFigure 6. IPTW-Adjusted Kaplan-Meier Curves Based on BMA Use in the AAP Cohort
eFigure 7. Differences in RMSTs Between BMA Users and Nonusers in the AAP Cohort
eFigure 8. IPTW-Adjusted Kaplan-Meier Curves Based on BMA Use in the ADT Cohort
eFigure 9. Differences in RMSTs Between BMA Users and Nonusers in the ADT Cohort

This supplementary material has been provided by the authors to give readers additional information about their work.

© 2024 Fukuokaya W et al. *JAMA Network Open*.

eFigure 1. Study Flow

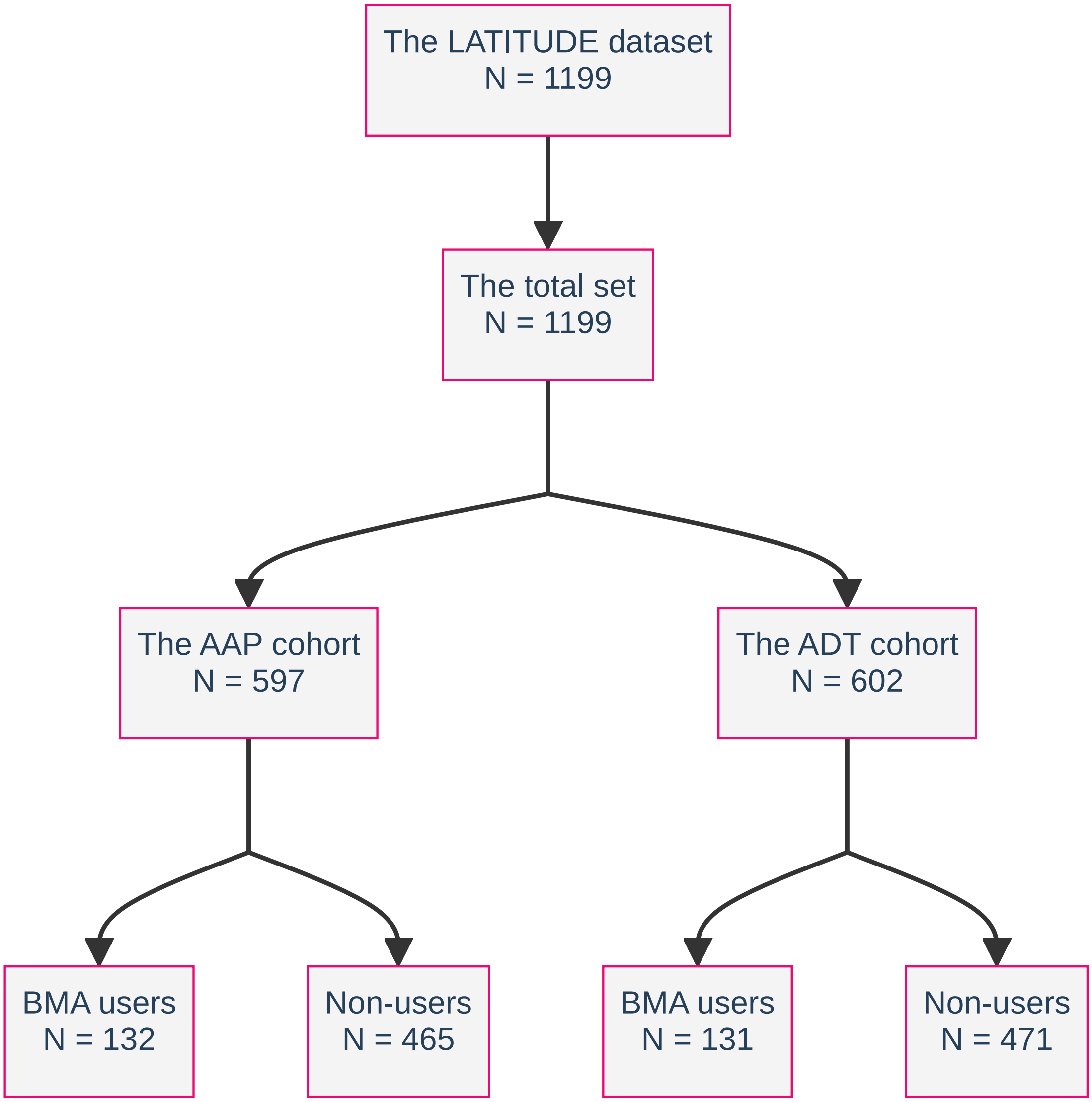

© 2024 Fukuokaya W et al. *JAMA Network Open*.

eFigure 2. Distributions of the Days Initiating BMA Based on Treatment Received

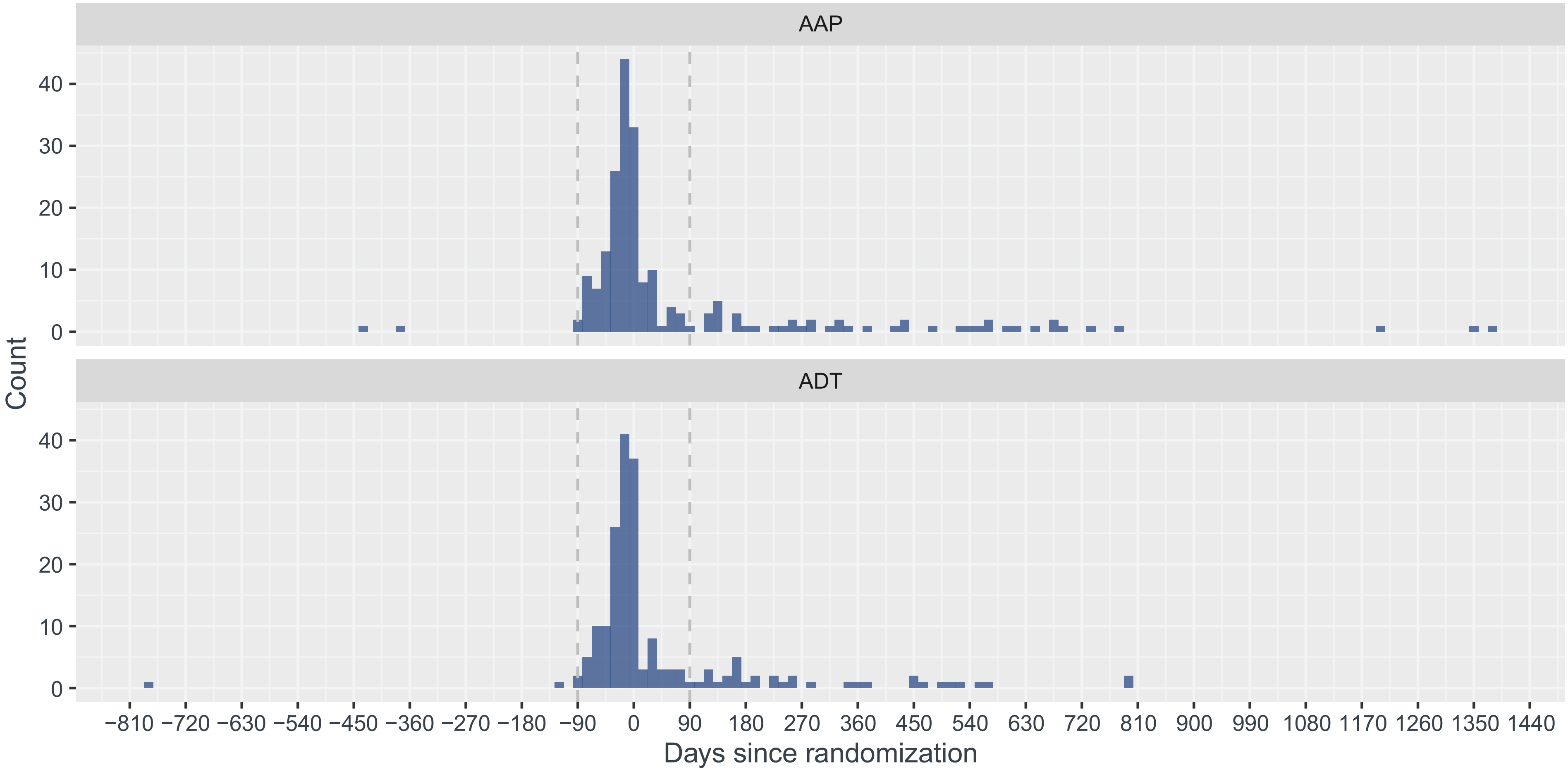

© 2024 Fukuokaya W et al. *JAMA Network Open*.

eFigure 3. Propensity Score Distributions Between BMA Users and Nonusers Before and After IPTW Adjustment in the Total Cohort

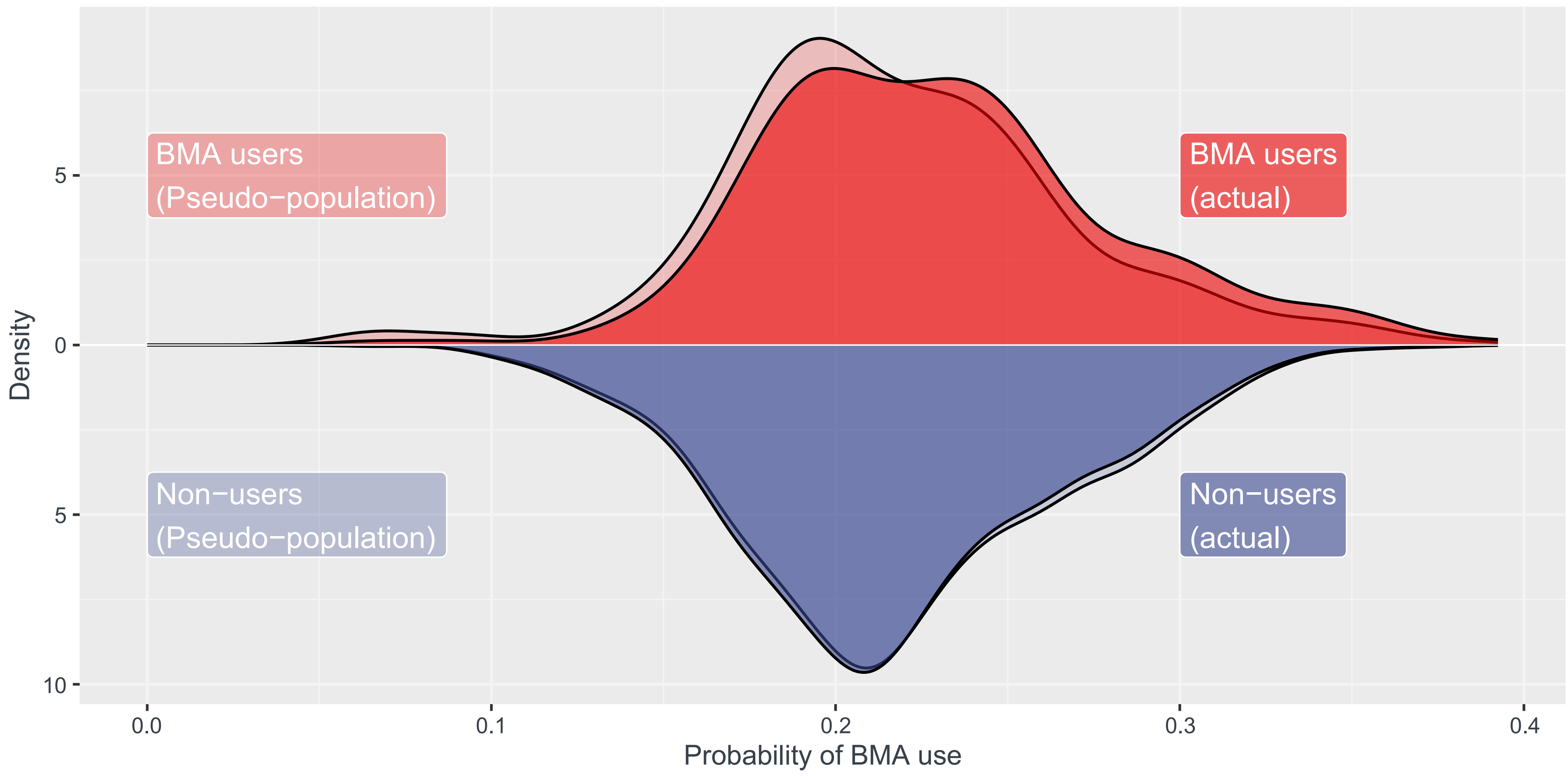

© 2024 Fukuokaya W et al. *JAMA Network Open*.

eFigure 4. Propensity Score Distributions Between BMA Users and Nonusers Before and After IPTW Adjustment in the AAP Cohort

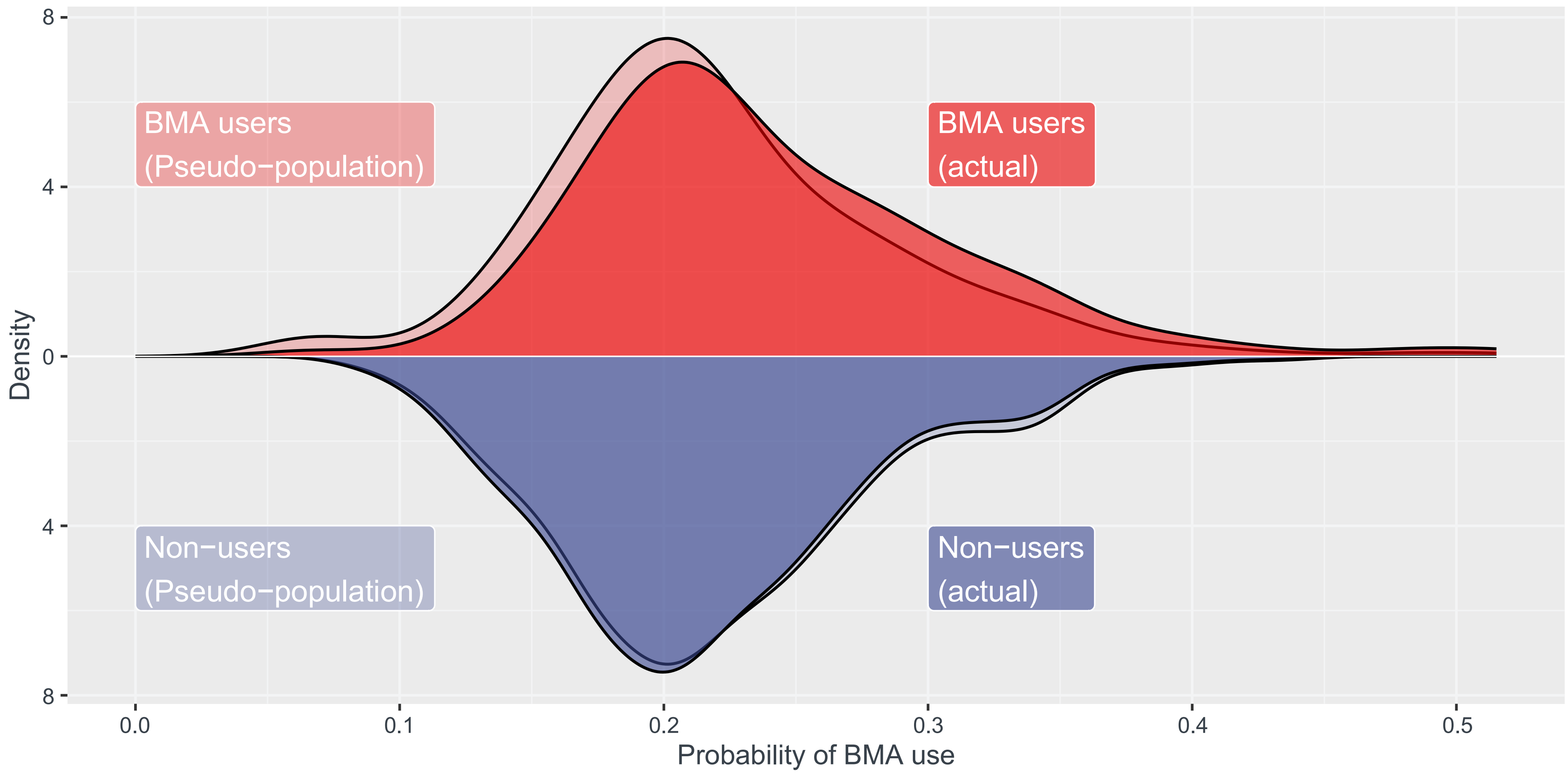

© 2024 Fukuokaya W et al. *JAMA Network Open*.

eFigure 5. Propensity Score Distributions Between BMA Users and Nonusers Before and After IPTW Adjustment in the ADT Cohort

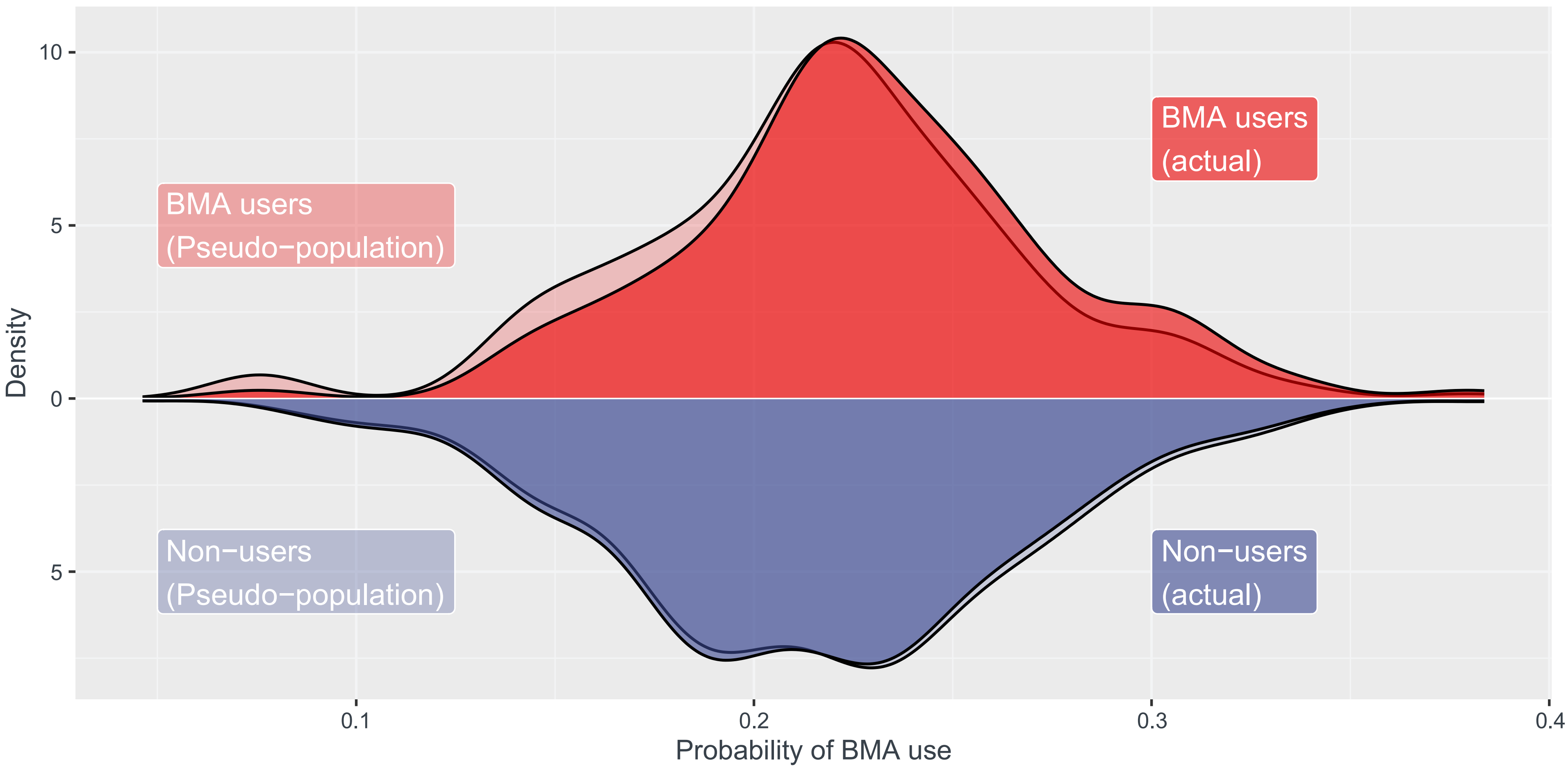

© 2024 Fukuokaya W et al. *JAMA Network Open*.

eFigure 6. IPTW-Adjusted Kaplan-Meier Curves Based on BMA Use in the AAP Cohort

Time to palliative radiation to bone
IPTW−adjusted, the AAP cohort

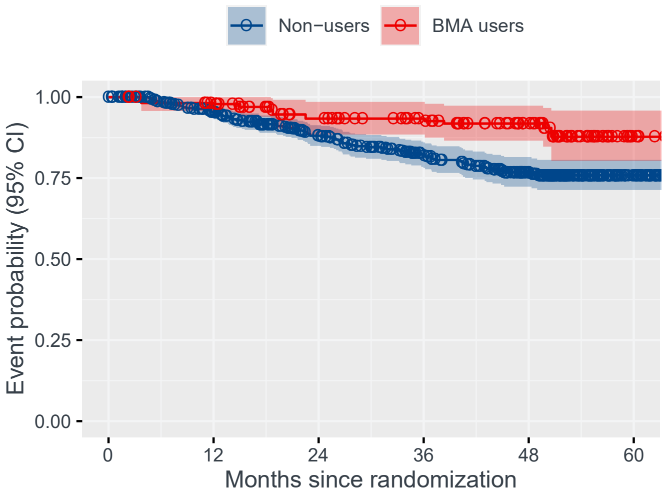

Number at risk

465	391	307	240	165	31
133	123	100	84	59	8

Time to clinical fracture
IPTW−adjusted, the AAP cohort

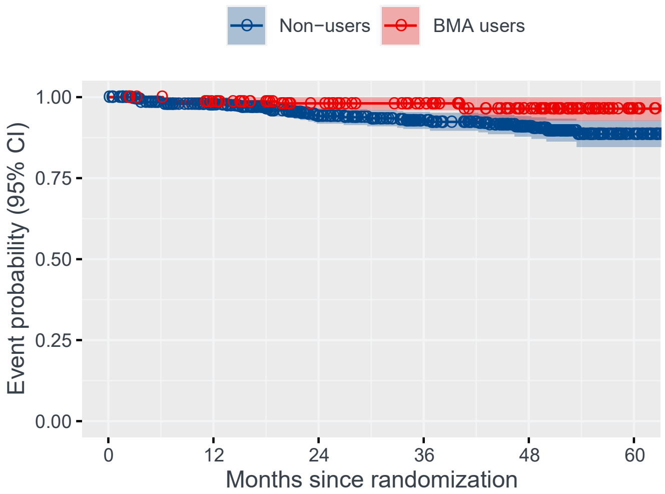

Number at risk

465	396	305	245	163	28
133	123	103	85	58	9

Time to spinal cord compression
IPTW−adjusted, the AAP cohort

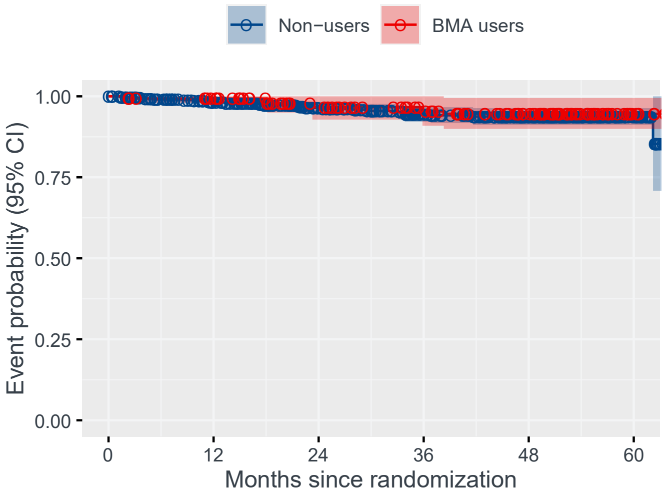

Number at risk

465	400	320	259	178	33
133	125	102	84	60	9

Time to surgery to bone
IPTW−adjusted, the AAP cohort

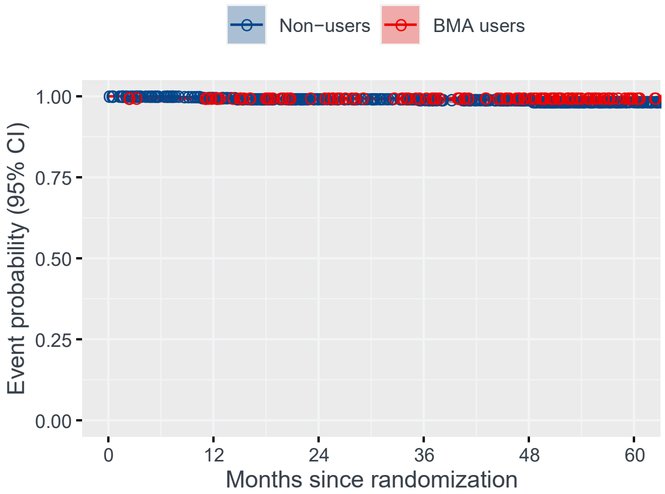

Number at risk

465	403	321	263	180	33
133	125	105	87	60	9

© 2024 Fukuokaya W et al. *JAMA Network Open*.

eFigure 7. Differences in RMSTs Between BMA Users and Nonusers in the AAP Cohort

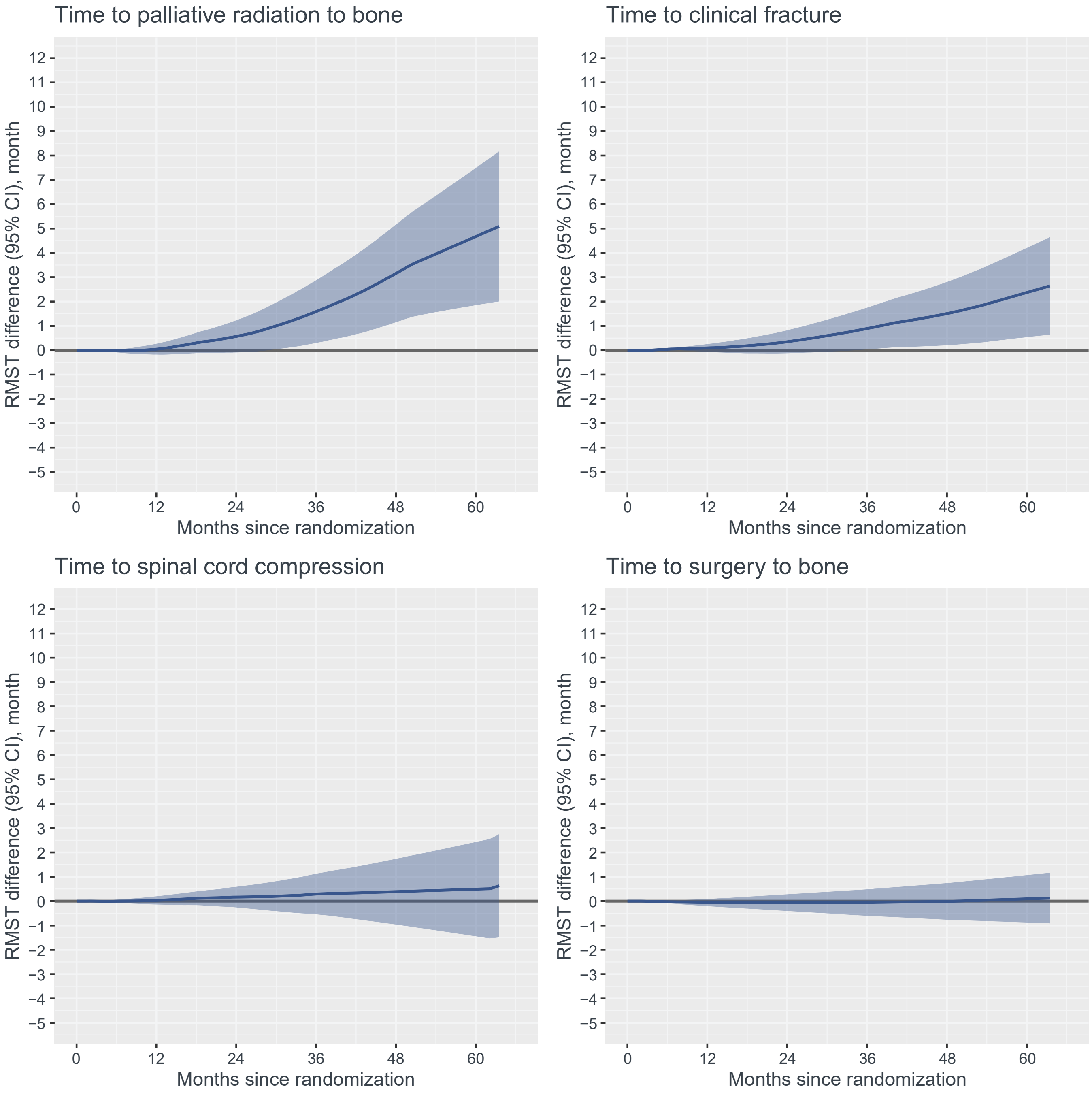

© 2024 Fukuokaya W et al. *JAMA Network Open*.

eFigure 8. IPTW-Adjusted Kaplan-Meier Curves Based on BMA Use in the ADT Cohort

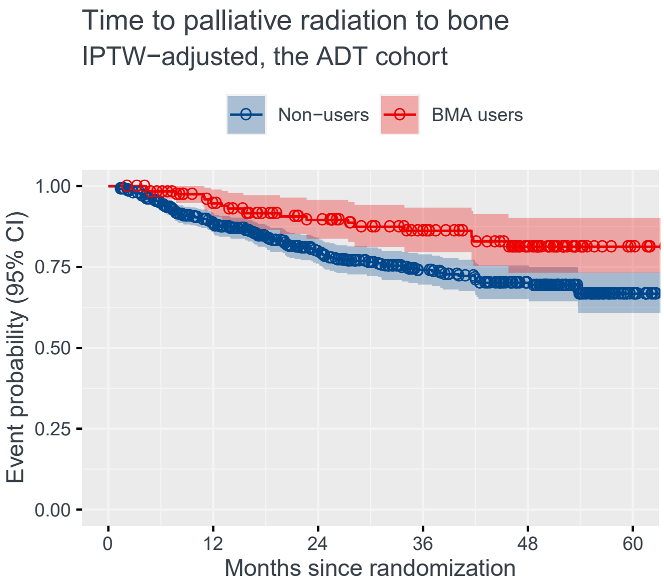

Number at risk

471	358	245	162	104	14
131	109	85	64	40	6

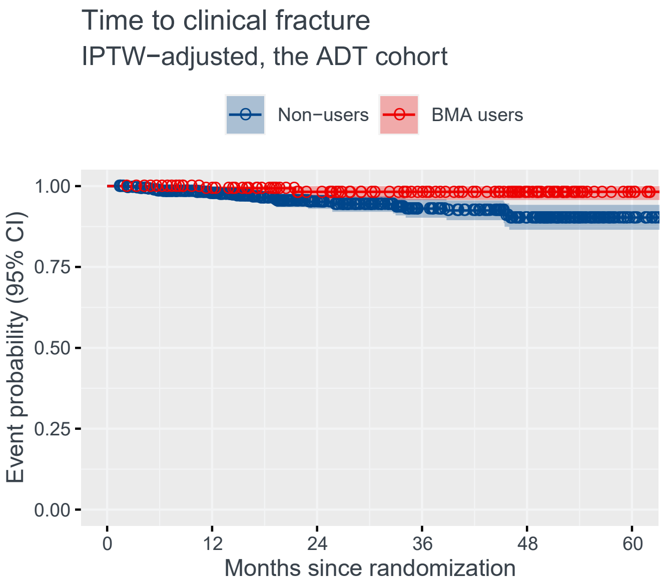

Number at risk

471	383	266	174	111	16
131	113	88	67	43	7

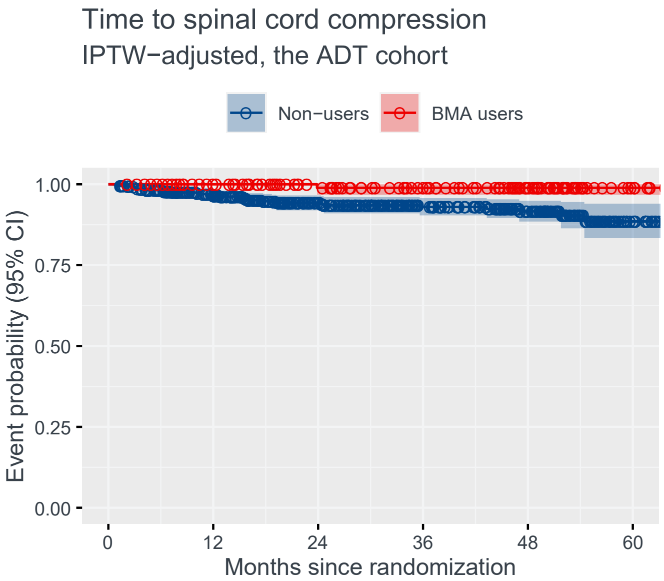

Number at risk

471	382	272	181	116	15
131	114	89	68	44	7

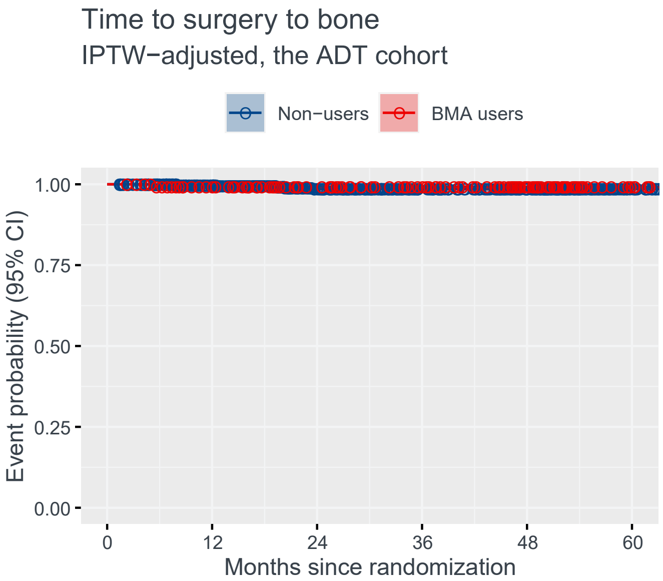

Number at risk

471	387	271	182	118	16
131	113	89	69	44	7

© 2024 Fukuokaya W et al. *JAMA Network Open*.

eFigure 9. Differences in RMSTs Between BMA Users and Nonusers in the ADT Cohort

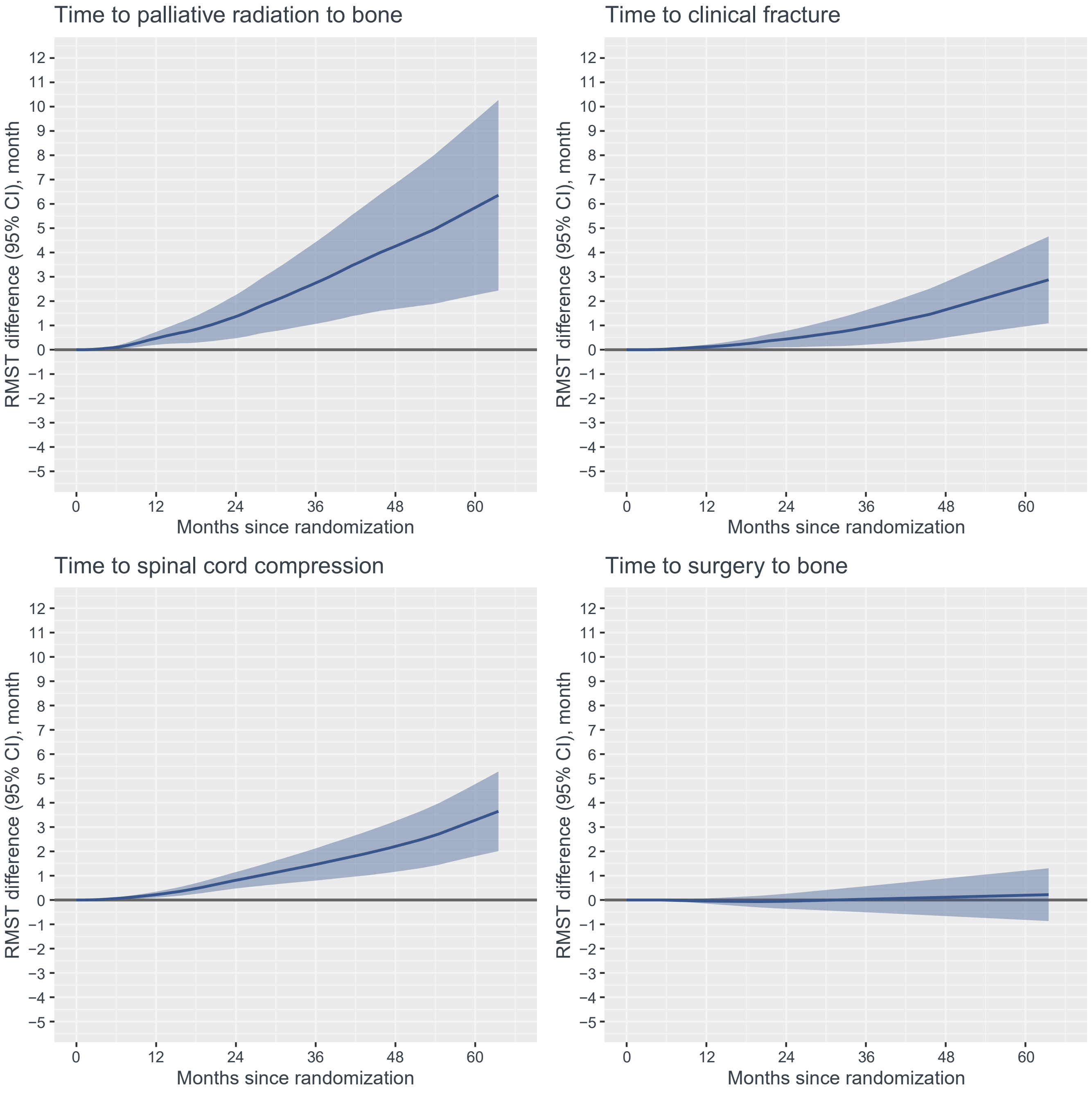

© 2024 Fukuokaya W et al. *JAMA Network Open*.
