# Supplementary material for: Bone-Modifying Agents in Patients With High-Risk Metastatic Castration-Sensitive Prostate Cancer Treated With Abiraterone Acetate
Source: JAMA Netw Open. 2024 Mar 15;7(3):e242467. doi: 10.1001/jamanetworkopen.2024.2467 (PMC10943414; doi:10.1001/jamanetworkopen.2024.2467)
Supplement: Supplement 3. — Data Sharing Statement [file jamanetwopen-e242467-s003.pdf]

Data Sharing Statement

Fukuokaya. Bone-Modifying Agents in Patients With High-Risk Metastatic Castration-Sensitive Prostate Cancer Treated With Abiraterone Acetate. *JAMA Netw Open*. Published March 15, 2024. doi:10.1001/jamanetworkopen.2024.2467

Data
Data available: No

Additional Information
Explanation for why data not available: Data are available at https://yoda.yale.edu/.
